# An unusual combination of a bilateral aberrant suprascapular artery with neurovascular structures variants

**DOI:** 10.1007/s00276-023-03157-0

**Published:** 2023-05-10

**Authors:** Maria Piagkou, George Tsakotos, Dimitrios Chytas, Trifon Totlis, George Triantafyllou, Nikitas-Apollon Panagiotopoulos, Athina Tousia, Konstantinos Natsis

**Affiliations:** 1 Department of Anatomy, School of Medicine, National and Kapodistrian University of Athens, 75 Mikras Asias Str., Goudi, 11527 Athens, Greece; 2 Basic Sciences Laboratory, Department of Physiotherapy, University of Peloponnese, Tripoli, Sparta Greece; 3 School of Medicine, European University Cyprus, Nicosia, Cyprus; 4 Department of Anatomy and Surgical Anatomy, School of Medicine, Faculty of Health Sciences, Aristotle University of Thessaloniki, Thessaloniki, Greece; 5 Department of Forensic Medicine and Toxicology, School of Medicine, National and Kapodistrian University of Athens, Athens, Greece

**Keywords:** Suprascapular artery, Variation, Origin, Axillary artery, Subclavian artery, Lateral thoracic artery, Subscapular trunk

## Abstract

**Purpose:**

The report describes a bilateral suprascapular artery (SPSA) of atypical origin in coexistence with neurovascular aberrant structures.

**Methods:**

The variants were identified in a 91-year-old formalin-embalmed male cadaver, derived from a body donation program after a signed informed consent.

**Results:**

The left-sided SPSA emanated from the 1st part of the axillary artery, coursed between the brachial plexus lateral and medial cords, accompanied by the suprascapular nerve, and passed below the superior transverse scapular ligament. Ipsilateral coexisted variants were the lateral thoracic artery multiplication, the subscapular trunk formation, and the musculocutaneous nerve duplication. In the right supraclavicular area, a SPSA duplication was identified. The main artery emanated from the thyrocervical trunk in common with the transverse cervical artery and the accessory SPSA emanated from the dorsal scapular artery. Both SPSAs coursed over the superior transverse scapular ligament, while the suprascapular nerve ran below the ligament.

**Conclusions:**

The current study reported a bilateral aberrant SPSA, originating from the AA 1st part (left side) and from the dorsal scapular artery (right side), which coexisted with adjacent neurovascular structures’ variants. The left SPSA atypically coursed below the superior transverse scapular ligament. Such an unusual combination of variations, present bilaterally in the current study, may be challenging for radiologists and surgeons.

## Introduction

The axillary artery (AA) typical, six-branch pattern is encountered in a quite low prevalence (27%) [8], fact that highlights the wide AA variability. The typical pattern includes the superior thoracic artery (occasionally absent), the thoracoacromial trunk, the lateral thoracic artery (LTA, occasionally multiplied), the subscapular artery (occasionally fused with adjacent arteries into the subscapular trunk of variable form), and the anterior and posterior circumflex humeral arteries (occasionally fused) [22]. Supernumerary branches may also occur isolated or fused into common trunks or emanating in common with the constant vessels [8]. Rarely, some of the subclavian artery branches may originate from the AA. Such an example is the suprascapular artery (SPSA) [7, 17] that typically emanates from the thyrocervical trunk, independently or by a common trunk with the transverse cervical artery (75%) [22]. Usually, it courses inferior to the transverse cervical artery, across the anterior scalene muscle and the phrenic nerve, posterior to the sternocleidomastoid muscle and the internal jugular vein. Thereafter, it continues laterally posterior to the subclavius muscle, crossing the subclavian artery and brachial plexus. The SPSA usually passes over the superior transverse scapular ligament (STSL) and reaches the supraspinatus fossa of the scapula. The suprascapular vein (SPSV) that accompanies the artery usually lies ventral and superior to it [22]. The SPSA may occur with a variant origin, course and termination, and coexist with variations in the adjacent structures.

The current cadaveric report describes an unusual, bilateral combination of anatomical variants. A bilateral SPSA aberrant origin from the AA 1st part (left side) and from the dorsal scapular artery (right side) was identified. The SPSAs of atypical course and termination, coexisted with adjacent neurovascular structures’ variants.

## Case report

**Fig. 1:**
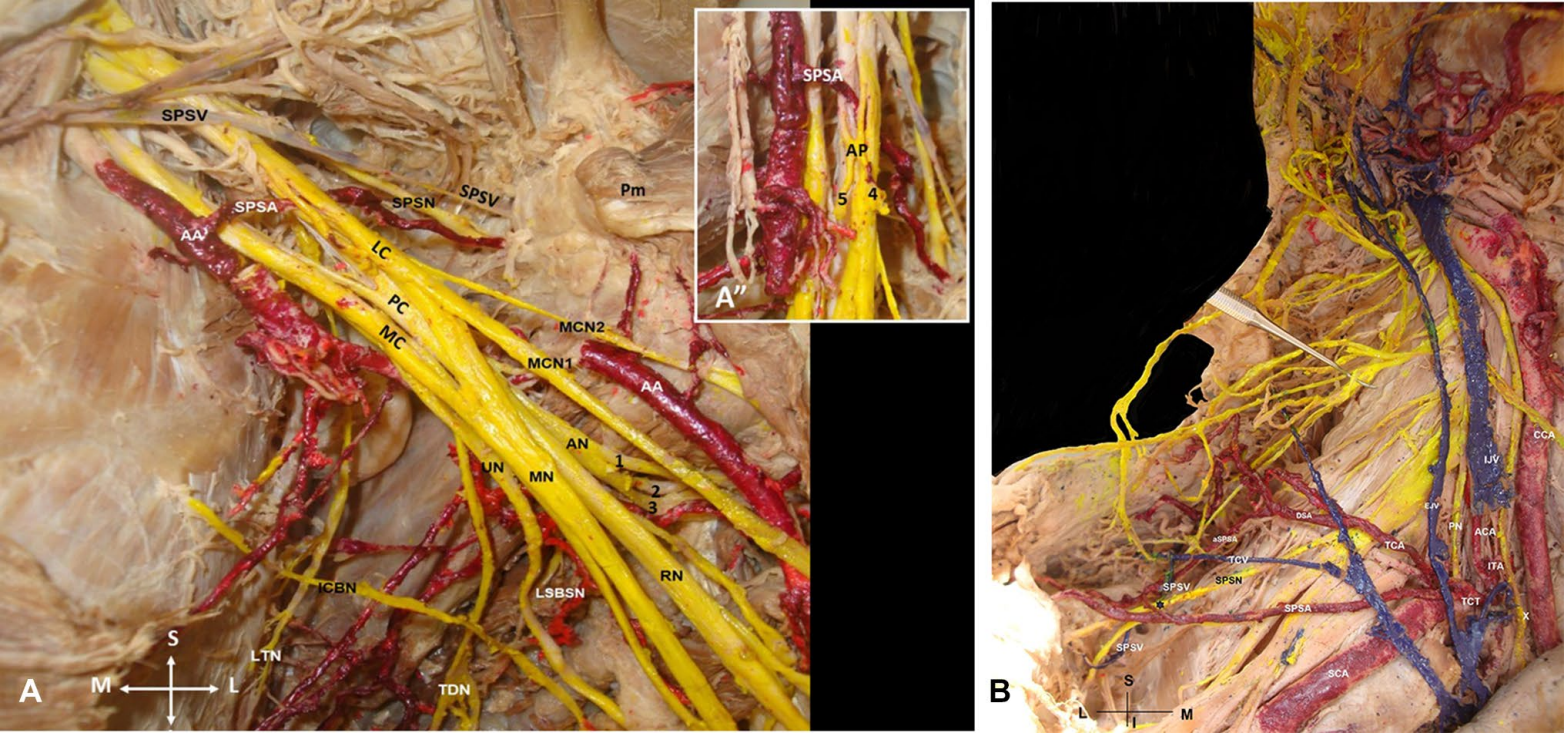
**A** Left and **B** Right axilla’s dissection. **A** Suprascapular vein (SPSV) passing over and suprascapular nerve and artery (SPSN and SPSA) coursing inferiorly to the superior transverse scapular ligament (STSL). The SPSA originated from the 1st part of the axillary artery (AA). The SPSA atypical course anterior to the medial cord (MC) and posterior to the lateral cord (LC) of the brachial plexus. The musculocutaneous nerve (MCN) duplication (1, 2), AN-axillary nerve trifurcation (1, 2, 3), RN-radial nerve, MN-median nerve, UN-ulnar nerve, TDN-thoracodorsal nerve, ICBN-intercostobrachial nerve, LTN-long thoracic nerve, LSBSN-lesser subscapular nerve, PC-posterior cord, **A**’’A zoomed view of the ansa pectoralis formation (AP), 4 and 5- lateral and medial pectoral branches, Pm- pectoralis minor, M-medial, L-lateral, S-superior and I-inferior orientation view. **B** Right supraclavicular area dissection, TCT-thyrocervical trunk giving rise to the ascending cervical artery (ACA), the inferior thyroid artery (ITA), and the common trunk of the main SPSA and the transverse cervical artery (TCA). TCA giving off the dorsal scapular artery (DSA). The accessory SPSA (aSPSA) emanated from the DSA and anastomosed with the SPSA (black asterisk). Both SPSAs passed over the STSL (lateral and medial termination points), *SPSV* suprascapular vein from the transverse scapular vein (TCV) passing inferior to the STSL, *CCA* common carotid artery, X-vagus nerve, *PN* phrenic nerve, *EJV* external jugular vein, *IJV* internal jugular vein and *SCA* subclavian artery, *M* medial, *L* lateral, *S* superior, and *I* inferior orientation view

During dissection of a formalin-embalmed 91-year-old male donated cadaver, a bilateral aberrant SPSA was identified. The subject donated his body before death (*from cachexia due to the Alzheimer disease*) to the Anatomy Department of the Medical School of the National and Kapodistrian University of Athens (NKUA), through the “Anatomical Gift Program” after a written informed consent. Details of the medical record of the subject were unknown. In the left axilla, at the 1st rib inferior border, the AA gave off an aberrant SPSA (atypically coursing between the brachial plexus lateral and medial cords, posterior to the ansa pectoralis formation) and a superior thoracic artery. The aberrant SPSA and the suprascapular nerve (SPSN) coursed below the STSL. The SPSV ran over the ligament and emptied into the external jugular vein (Fig. 1A). In the right supraclavicular area, a SPSA duplication was identified. The main SPSA typically emanated from the thyrocervical trunk by a common trunk with the transverse cervical artery and coursed anterior to the brachial plexus lateral cord. At the lateral third of the clavicle, it crossed with the SPSN and passed over the STSL, accompanied by the SPSV and the accessory SPSA. The accessory SPSA originated from the dorsal scapular artery, which atypically originated from the transverse cervical artery. The two SPSAs anastomosed, 1.8 cm proximal to the STSL (Fig. 1B). The SPSN arose from the brachial plexus upper trunk, ran inferior to the SPSA, and passed below the STSL (Fig. 1B).

**Fig. 2:**
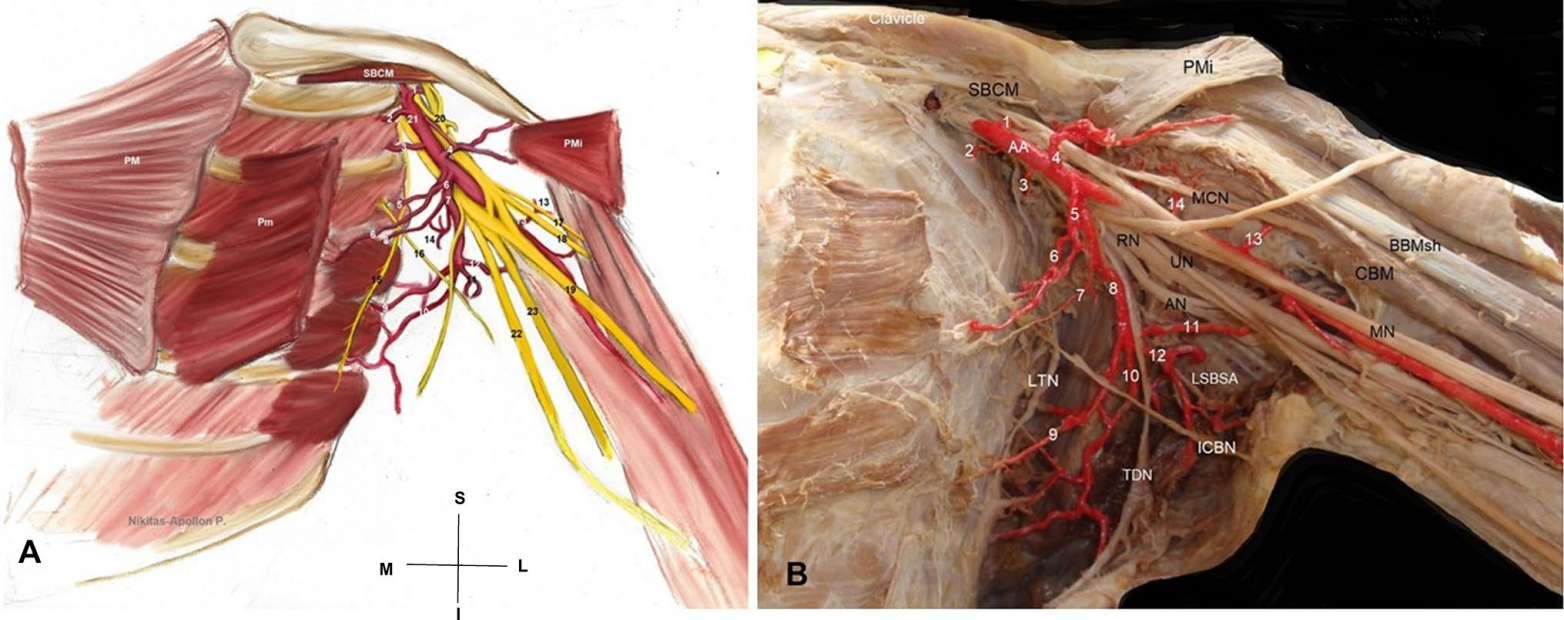
Left axilla dissection (**A** schematic representation and **B** cadaveric dissection). 1. The aberrant suprascapular artery, 2. Superior thoracic artery, 3. 1st lateral thoracic artery (LTA) from the axillary artery (AA), 4. Thoraco-acromial trunk, 6. 2nd LTA from the AA, 5. 3rd LTA from the subscapular trunk (SBST), 7. SBST, 8. 4th LTA from the SBST, 9. 5th LTA from the subscapular artery, 10. Thoracodorsal artery, 11. Circumflex scapular artery, 12. Posterior circumflex humeral artery, 13. Anterior circumflex humeral artery, 14. Muscular branches to the subscapularis upper part, 15. Long thoracic nerve, 16. Intercostobrachial nerve, 17 and 18. Musculocutaneous nerve duplication, 19. Median nerve, 20. Ansa pectoralis, 21. AA, 22. Ulnar nerve-UN, 23. Radial nerve-RN, SBCM-subclavius muscle, PM-pectoralis major, and Pm-pectoralis minor. **B** 5. SBST, 7. 2nd LTA from the AA, 6. 3rd LTA from the SBST, 8. SBST, 9. 4th LTA from the SBST, 11. Posterior circumflex humeral artery, 12. Common trunk of the circumflex scapular artery and the muscular branches to latissimus dorsi and teres minor muscles (lesser subscapular artery-LSBSA), 13. Anterior circumflex humeral artery, 14. Muscular branches to the coracobrachialis, *LTN* long thoracic nerve, *ICBN* intercostobrachial nerve, *MN* Median nerve, *PMi* pectoralis major insertion, *MCN* musculocutaneous nerve, *AN* axillary nerve, *CBM* coracobrachialis muscle, and *BBMsh* biceps brachii muscle short head, *M* medial, *L* lateral, *S* superior and *I* inferior orientation view

*Coexisted variants:* In the left axilla, two LTAs (the 1st and 2nd) emanated from the AA 2nd part. The subscapular trunk from the AA 3rd part, gave off the 3rd and 4th LTAs and the subscapular artery that divided into the 5th LTA, the thoracodorsal, the circumflex scapular and the posterior circumflex humeral artery. The anterior circumflex scapular artery directly emanated from the AA. The single intercostobrachial nerve coursed through the 3rd and 4th LTAs and the long thoracic nerve descended posterior to them. A MCN duplication was also identified. The main MCN had a typical course and branching pattern and the accessory one innervated coracobrachialis and the short head of the biceps brachii muscle (Fig. 2A, B). Overall, the cadaver presented no obesity or muscle atrophy. No signs of pathological conditions, trauma, or earlier surgery in the cadaver’s upper limbs were identified.

## Discussion

The current report describes an unusual, bilateral combination of anatomical variants. At first, we noted a bilateral aberrant SPSA originating from the AA 1st part (left side) and from the dorsal scapular artery (right side, the accessory vessel from the duplicated SPSA). The SPSA variant origin from the subclavian artery has been described by several authors [22, 25], and has been found to occur at a percentage of 2% [24], while the axillary origin has an incidence ranging between 1.6 and 3.8% [16, 17, 21]. Naidoo et al. [17] reported an axillary origin of the SPSA (from the 1st part in 2% bilaterally, from the 2nd part in 5% unilaterally). Other SPSA unusual origins include the internal thoracic artery (1–11%), the inferior thyroid artery (3.84%) [7], the costocervical trunk (1%) [21, 23, 25], the subclavian artery 1st part (1–2 mm lateral to the internal thoracic artery, a high origin) [5], and the dorsal scapular artery [21]. Bilateral aberrant origins from the AA 3rd part (distal origins) are uncommon [7, 14, 20], as well as the SPSA distal origin from the subscapular artery, while the artery course into the suprascapular notch has been described by many authors [1, 9–11, 19]. Polgui et al. [19] and Al-Redouan et al. [1] identified a course of SPSA under the SPSL at 12.3 and 26.7%, respectively. The SPSA subclavian origin, proximal to the internal thoracic artery, is challenging to dissect, and may cause postoperative ischemia [6]. Ferreira [7] described a SPSA duplication, with the accessory SPSA forming an anastomosis with the main SPSA, as in the current case. Moreover, in the current case, the SPSA atypically coursed at the left side between the brachial plexus lateral and medial cords, while at the right side, its course was typical, anterior to the brachial plexus, like in the majority of the published cases (71%) [4]. The SPSA atypically courses between the brachial plexus upper and middle trunks in 28% and posterior to the brachial plexus in 1% [4]. The SPSA typically passes over the STSL [22]. Its course inferior to the STSL is less common [23]. In the present case, both SPSA and SPSN terminated inferiorly to the STSL, at the left side.

Developmentally, the main vessels derive from a primary capillary plexus. Under prevailing conditions, some vessels enlarge and reach their definite form and others regress [2]. During process, the variant branching pattern, including both variant origin and/or course, may appear. The current report highlights the coexistence of a bilateral aberrant SPSA of atypical course and termination (unilaterally), a SPSA duplication (unilaterally), with five LTAs, a subscapular trunk and a double MCN (unilaterally). In addition, the atypical branches coexisted with the absence of other AA branches, such as the superior thoracic artery. The accessory LTAs emanated from both the AA and the subscapular trunk. In the literature, variant LTA origins include the thoracoacromial artery (67.62%), the axillary (17.02%), the thoracodorsal (5%), and the subscapular artery (3.93%) [13]. The LTAs multiplication (3.09%), as well as its variant origin is clinically important [13], since the artery should remain intact during neck and breast surgery [8]. The formation of common trunks among the AA branches is quite uncommon. The subscapular artery has been identified to originate in common with the LTA (28.7%), with the posterior circumflex humeral artery (15.2%), and with the LTA and posterior circumflex humeral artery (4.7%) [8]. In the present study, the accessory LTAs emerged both from the AA, the subscapular trunk, or the artery, similarly to Panagouli et al. [18] described case, in which the LTA emerged from the subscapular artery. The abnormal origin, course, and termination of the AA branching pattern may cause confusion in angiographic studies and complicate surgery [4]. In the current study, the main MCN had a typical course and branching pattern, while the accessory one (derived after the MCN duplication) innervated both coracobrachialis and the biceps brachii short head. In Miller and Trelease study [15], the duplicated MCN supplied the muscles of the anterior arm compartment and provided lateral antebrachial cutaneous innervation.

The SPSA is clinically important during surgery in the anterior neck and supraclavicular region and must be identified and ligated [12]. The artery’s injury may cause micro-embolic events at the suprascapular nerve small vessels leading to neuropathy [7]. The knowledge of the SPSA variants is clinically important in arthroscopic SPSN decompression, in management of glenohumeral region disease and acromioclavicular joint reconstruction [3, 16]. The meticulous knowledge of the origin and course of possible SPSA variants is of paramount importance [12]. The SPSΑ course below STSL (into the fibro-osseous tunnel) adjacent to the SPSN can reduce the amount of surface area, exerting pressure on the fragile nerve, causing neural micro-trauma, resulting in neuropathy [23].

## Conclusions

The current study reported a bilateral aberrant SPSA, originating from the AA 1st part (left side) and the dorsal scapular artery (right side), which coexisted with adjacent neurovascular structures’ variants. The left SPSA atypically coursed below the STSL. Such an unusual combination of variations, present bilaterally in our study, may be challenging for radiologists and surgeons.

## Data Availability

Data will be available with the corresponding author for further reference.
